# Social influence of e-cigarette smoking prevalence on smoking behaviours among high-school teenagers: Microsimulation experiments

**DOI:** 10.1371/journal.pone.0221557

**Published:** 2019-08-29

**Authors:** Dingding Chao, Hideki Hashimoto, Naoki Kondo

**Affiliations:** 1 Department of Health and Social Behavior, School of Public Health, The University of Tokyo, Tokyo, Japan; 2 Department of Systems Innovation, School of Engineering, The University of Tokyo, Tokyo, Japan; University of Toronto, CANADA

## Abstract

The prevalence of electronic cigarette (e-cigarette) use has rapidly increased among young people, while conventional cigarette use has decreased in this age group. However, some evidence suggests that e-cigarette use is likely to induce conventional cigarette smoking. The present study explored the social influence of the prevalence of e-cigarette use in the peer network and in the general population as a potential mechanism by which e-cigarette use affects adolescents’ overall smoking behaviours. For this purpose, we developed an agent-based model in which young agents repeatedly choose to smoke conventional cigarettes and/or e-cigarettes, or to remain non-smokers. The choice is based on the agent’s evaluation of the utility derived from smoking and attitude towards smoking (‘openness’), which is influenced by smoking prevalence in the agent’s peer network and in the broader society. We also assumed a ‘crossover’ effect between the different types of smoking. The model was calibrated with United States National Youth Tobacco Survey data to reflect real-world numbers. We further simulated the prevalence of different types of smoking under counterfactual scenarios with different levels of openness and crossover effects. The models developed successfully reproduced actual prevalence trends in different types of smoking from 2011 to 2014. Openness to smoking is associated with a dramatic increase in e-cigarette smoking and especially in dual smoking, which cancels out the decline in sole conventional smoking. Larger crossover effects are associated with a higher prevalence of conventional smoking. The simulation results indicate that the social influence of the prevalence of e-cigarette use may influence young people to initiate or continue conventional cigarette smoking. Assessing the impact of e-cigarettes in the general population as a ‘healthier’ alternative to conventional smoking may require carefully monitoring trends in young people’s smoking behaviours.

## Introduction

Electronic cigarettes (e-cigarettes) are a form of electronic delivery of tobacco-derived products containing nicotine that is rapidly obtaining popularity in the United States and other countries [1, 2]. Compared with the prevalence of conventional cigarette smoking, which is distributed evenly across age groups, the prevalence of e-cigarette use is disproportionally high among young people.

The latest Cochrane review on the topic concluded that e-cigarettes mimic and recreate the sensations of conventional smoking with less toxic chemical exposure, and a consensus is growing on the effectiveness of e-cigarette use for conventional smoking cessation, as a ‘healthier alternative’ among adult smokers [3]. However, the impact of e-cigarette use on the initiation of any type of smoking among children and young people remains highly contentious [4, 5].

Although the rapid rise in e-cigarette use has been paralleled by a decrease in conventional cigarette smoking, growing evidence suggests that the use of e-cigarettes may induce the initiation of conventional cigarette smoking among young people [6–11]. The Surgeon General’s Office reported that, whereas the sole use of conventional cigarettes declined among young people from 2011 to 2015 (before the Food and Drug Administration’s prohibition of e-cigarette sales to adolescents), the dual use of e-cigarettes and conventional cigarettes increased rapidly among this age group during the same period [1]. Other studies have shown that e-cigarette use may be associated with intention to smoke conventional cigarettes, especially among young people [12–17]. Although determining whether e-cigarette use will eventually replace or encourage conventional cigarette smoking in the long term is a public health concern, few studies have explored the mechanisms behind the association between e-cigarette use and conventional cigarette use among adolescents. A possible mechanism for the association involves the social influence of the peer network and the general population on teenagers’ attitudes towards smoking [4, 12, 14, 16].

Social influence is a factor that affects the initiation of smoking: Individuals’ behaviour is influenced by their peers and by other groups to which they are linked through social networks [18]. The increasing prevalence of e-cigarette use is likely influential in changing community norms and individuals’ attitudes towards the use of both e-cigarettes and conventional cigarettes. Some studies have indicated that exposure to e-cigarette use may be associated with intention to initiate conventional cigarette smoking [14, 15, 19] and other risky health behaviours such as alcohol and drug use [20, 21].

To quantitatively assess the net effect of this influence, we used an agent-based microsimulation model that allows exploration of an individual’s decision making under dynamic exposure to social influences. Agent-based modelling is ‘a computational approach in which agents with a specified set of characteristics interact with each other and with their environment according to predefined rules’; it has recently been applied to public health topics such as disease epidemics, health behaviours and social epidemiology [22]. Agent-based modelling provides a way to gain insight into a simple behavioural rule/mechanism through which population patterns arise and to implement counterfactual simulations to seek leverage points for policy intervention, which may be infeasible with real-world observations [23–25]. By focusing on social influence from the peer network and the surrounding society on attitudes towards smoking, the present study aimed to simulate how the perceived acceptance of the use of conventional cigarettes and e-cigarettes among teenagers in high school and the surrounding society affects the prevalence of each type of smoking, using hypothetical experiments.

## Materials and methods

### Description of the model

For more details about the characteristics of agent-based modelling compared with other existing approaches and our rationale for adopting agent-based modelling for this study specifically, refer to Section 1 in S1 Appendix.

**Fig 1:**
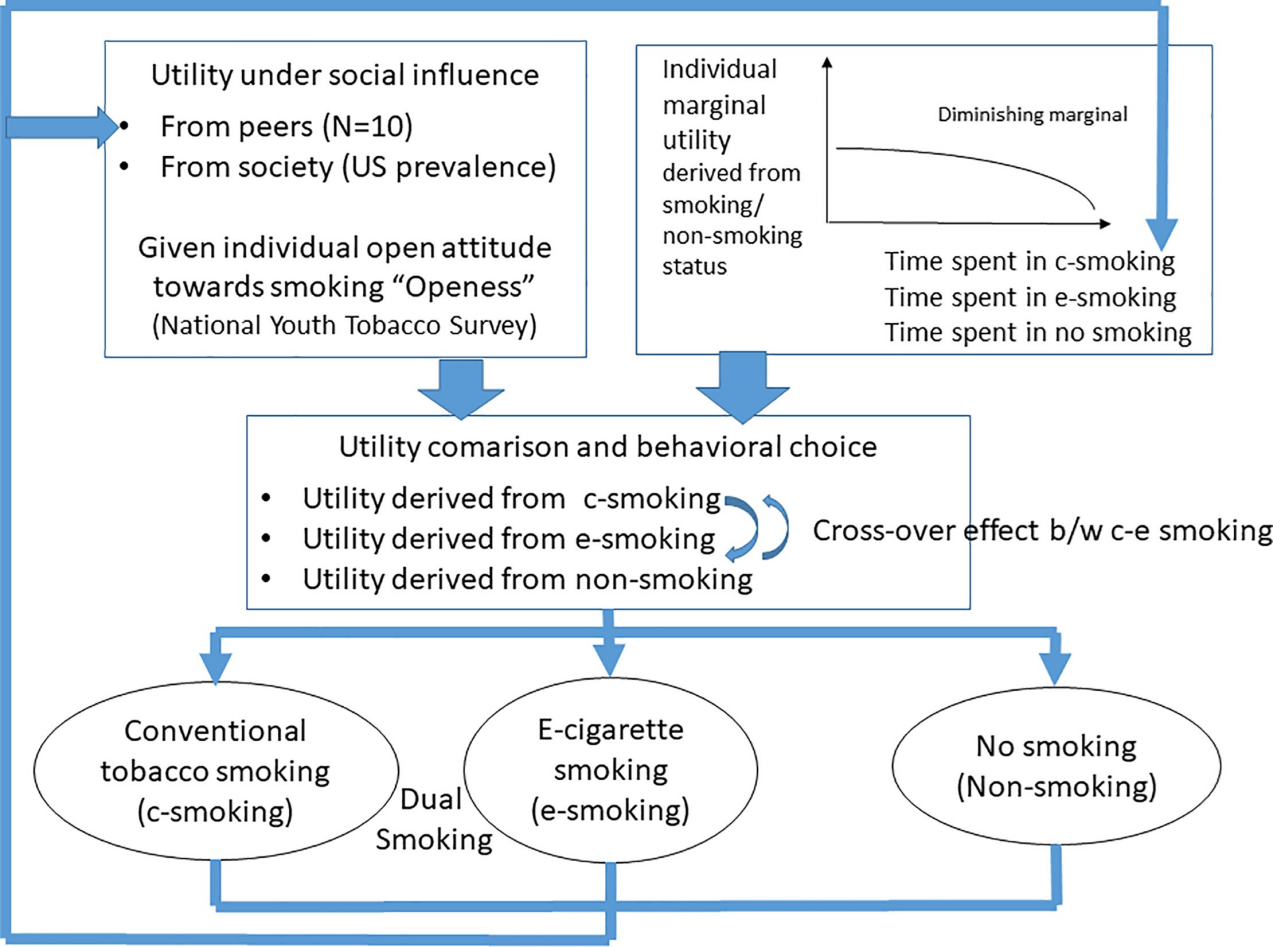
Model description. Cycle of behavioural choice for smoking status and types of smoking.

In this study, we designed a decision model for the agent (teenager in high school), as depicted in Fig 1. In this model, each agent is supposed to decide his/her smoking/non-smoking behaviour based on the utility calculation summing his/her own marginal utility derived from smoking/non-smoking status and the utility under social influence from his/her close peers and from society as a whole. Peer social influence is parameterised by referring to the number of smokers in the agent’s close network, and societal influence is parameterised according to the prevalence of each smoking type in the United States. The young agents are influenced differently depending on their levels of open attitudes towards the different types of smoking. After the behavioural decision, the agent’s own behavioural status and that of his/her close network are revised for the next cycle of decision making, the revised behavioural statuses of the agents are fed back into the model to reshape a revised social influence environment and the cycle is repeated. After a validity check of the model through a comparison with the actual trend change in teenagers’ smoking prevalence, we modified key parameters related to the agent’s vulnerability to social influence to determine how the prevalence rates of the different types of smoking affect each other through agents’ behavioural decisions.

More specifically, we assumed that young agents make decisions to start, continue or stop smoking conventional cigarettes and/or e-cigarettes according to the ‘utility’ calculation described below. (For a detailed description of the theoretical background of this utility-based model, refer to Section 2.1 in S1 Appendix). We assumed that these agents would make decisions based on their own perceptions about smoking, while also being susceptible to social influence from their peer networks and from society in general.

To model these relationships, we developed an agent-based model based on the 2011–2014 National Youth Tobacco Survey [26]. The model contained a scale-free network of 3000 high school teenager agents. We determined the initial distribution of the smoking statuses of the agents using the percentage of current use (last 30 days) of conventional cigarettes or e-cigarettes among United States high school students, based on 2011 National Youth Tobacco Survey data [26]. We then generated a network in which agents preferred to connect to other agents who already had more connections [27]. The young agents were connected to a certain number (*n*) of ‘smoker’ agents, based on their response to the question ‘How many of your four closest friends smoke cigarettes?’

In each decision cycle, the agents evaluated the utility derived from potential alternative choices of smoking conventional cigarettes and/or using e-cigarettes or remaining a non-smoker, selected the alternative with the highest utility and updated their smoking status for the next cycle. The utility function for smoking included two major components: the agents’ own smoking experiences based on their frequency of smoking and the social influence of the vicarious experience of others in the agents’ network and in society as a whole.

We assumed that social influence was realised through agents’ perceptions of the social popularity of smoking, or smoking prevalence in their peer network and in the United States in general. We then modelled how that perceived social influence would affect agents’ ‘openness to smoking’, or tendency to accept smoking. We quantified this tendency using the responses to the following questions: ‘If one of your friends offered you a cigarette, would you smoke it?’ and ‘Do you think you will smoke a cigarette anytime during the next year?’

Furthermore, we incorporated ‘*crossover*’ to represent a possible interaction between the prevalence of e-cigarette use and the prevalence of conventional cigarette use. In the baseline scenario, we assumed ‘crossover’ = 0, indicating that the influences of e-cigarette use and conventional cigarette use are independent. The details of the model are included in Section 2.3 in S1 Appendix, S1 Table and S2 Table.

### Model calibration

The agents’ initial profiles (e.g. age, number of friends who smoke, openness to smoking and smoking status) were generated based on United States data from the 2011 National Youth Tobacco Survey [26]. Respondents who did not answer the relevant questions were excluded from the generation of the profiles. We developed both the model and the simulation using NetLogo 5.3.1, and we present the algorithm used in S2 Appendix.

We calibrated the model to fit the smoking prevalence in the 2011 National Youth Tobacco Survey and conducted backward validation by comparing the simulation results with the actual prevalence trends of conventional cigarette and e-cigarette use from 2012 to 2014. We did not include data from the 2015 survey, which reported a dramatic change in the prevalence of e-cigarette use, presumably because of the United States Food and Drug Administration’s new ban on the sale of e-cigarettes to children aged under 18 years [28].

### Simulation scenarios

Based on the baseline model developed above (Scenario 0, ‘openness’ = 0 and ‘crossover’ = 0), we explored several counterfactual scenarios to examine how agents’ attitudes and social influence on conventional cigarette use and e-cigarette use interactively affect their prevalence rates. More specifically, we incrementally increased the magnitude of agents’ openness to smoking by 0.1 (‘*openessCC*’ and ‘*openessEC*’ in the Section 2.3 in S1 Appendix) from 0 to 0.3. We also modified ‘*crossover*’ incrementally from 0 to 0.3. We ran the simulations 100 times for each scenario and recorded the average results.

### Ethical statement

Because this study used publicly available, anonymised data, as described above, ethical consent requirements were not relevant.

## Results

**Fig 2:**
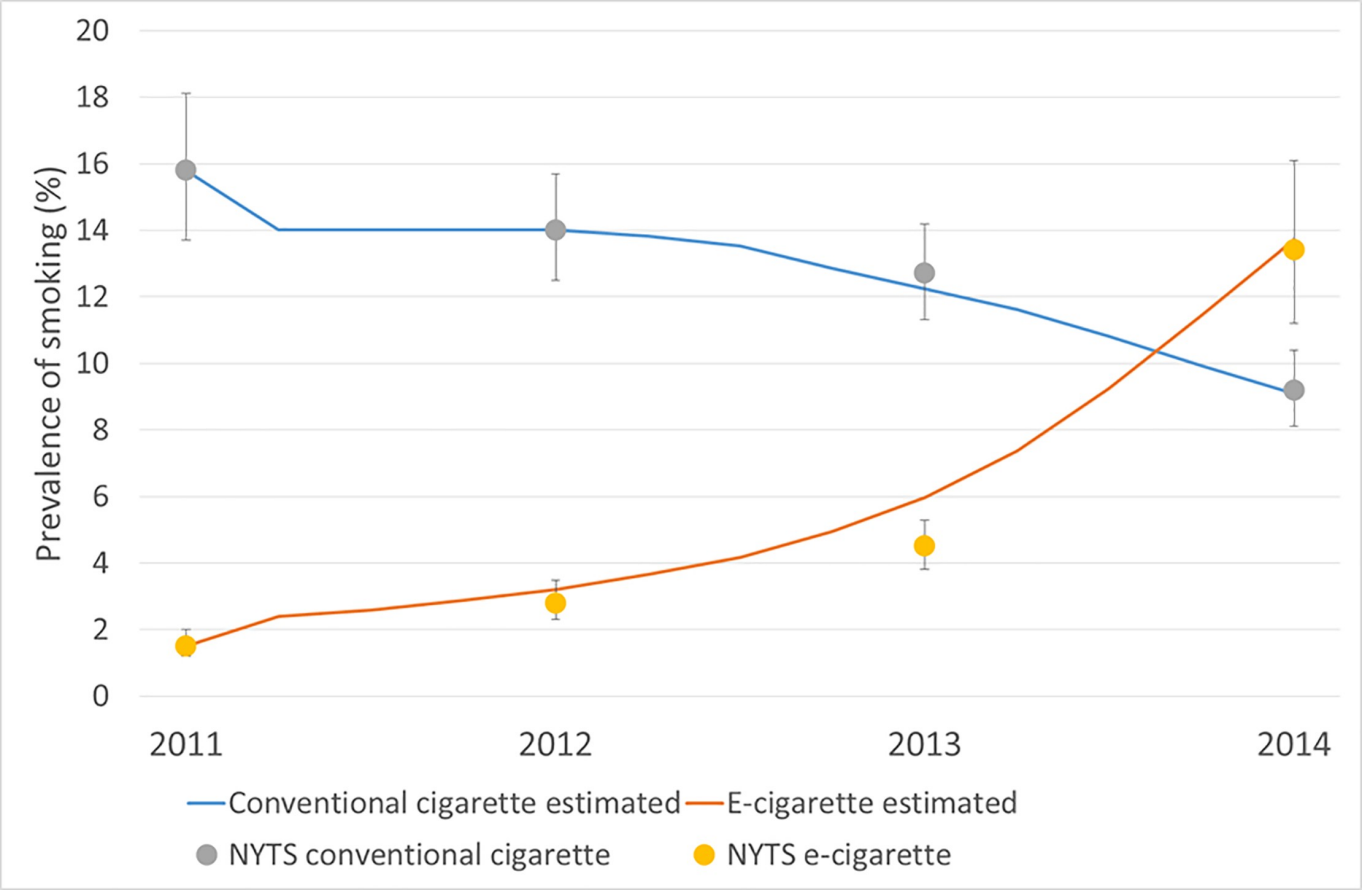
Simulated prevalence rates by type of smoking (lined) compared with the actual trends (dot) among teenagers in high school in the United States, 2011–2014. For each included year, United States National Youth Tobacco Survey data were referred to for the actual prevalence rates and their 95% confidence intervals.

Fig 2 shows the simulation results for the prevalence rates of the different types of smoking, compared with the actual trends observed from 2011 to 2014. Note that the prevalence rates of conventional cigarette and e-cigarette use included dual smokers. Except for 2013, the estimated numbers were within the 95% confidence interval ranges of the reported prevalence rates, suggesting that our model successfully reproduced the actual prevalence trends in conventional cigarette, e-cigarette and dual smoking behaviours among teenagers in high school in the United States.

**Fig 3:**
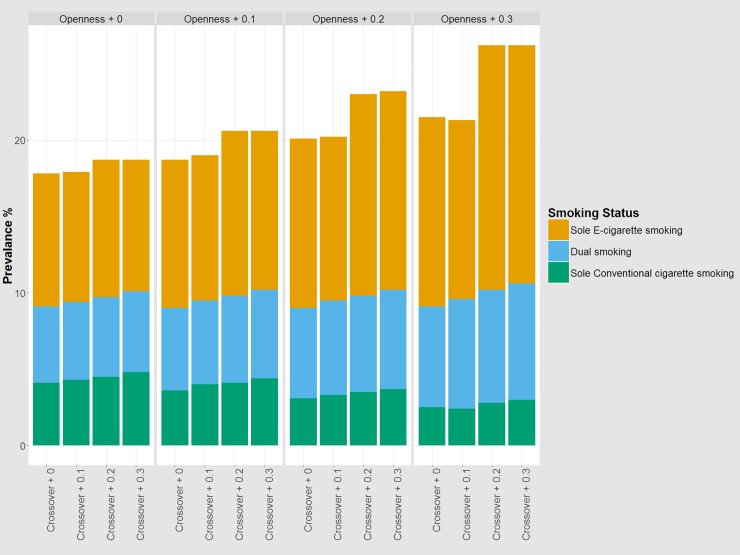
Simulated prevalence rates by type of smoking in counterfactual alternative scenarios as of 2014. ‘Openness + 0’ and ‘Crossover + 0’ represent the baseline scenarios and reflect real-world data drawn from the United States National Youth Tobacco Survey in 2014, as depicted in Fig 1. Simulation results of counterfactual scenarios with incrementally increased levels of young people’s openness to smoking and crossover effects between modes of smoking are presented.

Fig 3 shows the baseline prevalence rates of different smoking types in 2014 (openness + 0 and crossover + 0) and the simulation results under counterfactual scenarios with different levels of ‘openness’ and ‘crossover’. At baseline, the prevalence was 4.1% for sole conventional smoking, 8.7% for sole e-cigarette smoking and 5.0% for dual smoking of conventional cigarettes and e-cigarettes.

Compared with the baseline, an increase in ‘openness’ had a considerable impact on the prevalence of sole e-cigarette smoking: Given zero crossover effect, the higher level of ‘openness’ would lead to increases in the prevalence rates of e-cigarette smoking and dual smoking (to 12.4% and 6.6%, respectively), but to a decrease in the prevalence of sole conventional smoking (to 2.5%). The increase in dual smokers would offset the decrease in smokers of only conventional cigarettes, resulting in a persistent prevalence of conventional cigarette smoking despite the increased popularity of e-cigarette smoking.

In contrast, an increase in ‘crossover’ would result in a marginal increase in the prevalence of conventional cigarette smoking and a marginal decrease in the prevalence of e-cigarette smoking. Given zero ‘openness’, as in the baseline case, an increase of ‘crossover’ from 0 to 0.3 would lead to a 4.8% prevalence of sole conventional smoking, a 8.6% prevalence of sole e-cigarette use and a 5.3% prevalence of dual smoking. The increase in ‘crossover’ would result in a slight net increase in the prevalence of conventional cigarette smoking.

## Discussion

There has been a great deal of debate on the safety of e-cigarette use, and a consensus has recently been reached that e-cigarette use at least poses a lower risk than does conventional cigarette smoking [3, 29, 30]. However, even if e-cigarette use is a lower-risk alternative, it remains questionable whether it can eventually replace conventional cigarette smoking. The most recent observational study in the United States indicated that adult e-cigarette users were significantly more successful in smoking cessation, although this previous study’s data may have been contaminated by a concurrent anti-tobacco media campaign and the selective use of e-cigarettes by those with stronger intentions to quit [31]. Furthermore, the impact of e-cigarette use on young people who are at risk of tobacco initiation requires independent research [32].

Previous simulation results have consistently indicated that the prevalence of e-cigarette use will increase rapidly [25, 33, 34]. Our simulation model provides insight into the mechanism by which young people’s open attitudes to e-cigarette use and the prevailing popularity of e-cigarettes in their local networks and in the broader society can be strong drivers for rapid and massive prevalence increases.

It is noteworthy that our simulation with the condition of ‘crossover’ = 0 (assuming no crossover effect between conventional cigarette smoking and e-cigarette use) indicated that, despite the rapid increase in e-cigarette use, the net number of conventional cigarette smokers will remain almost unchanged because of the increase in the number of dual smokers. This is because young conventional cigarette smokers are more likely to become dual smokers than to switch to e-cigarette smoking only, and some portion of e-cigarette initiators also become dual smokers. This phenomenon may be explained by the findings of previous studies showing that young people are motivated to start using e-cigarettes by curiosity, rather than by the intention to quit smoking [11–13]. Our simulation fits real-world observations of a slowing down of the decline in conventional cigarette use and an increase in dual smokers from 2014 to 2015 among young people in the United States [1].

Our findings further indicate that, among young people, if the influence of e-cigarette use on the initiation of conventional cigarette smoking (crossover) increases, an increase in the prevalence of e-cigarette smoking in society will be accompanied by an increase in dual smoking and, ultimately, also an increase in conventional cigarette smoking. These findings suggest that policies aimed at encouraging e-cigarette use as a healthier alternative to conventional cigarette smoking among adults may need careful re-examination to prevent conventional tobacco smoking among young people.

A simulation study conducted by Levy et al. [33] suggested that the impact of e-cigarettes depends on the prevalence rates of e-cigarette use and dual smoking, which is in line with our findings. Furthermore, a simulation by Kalkhoran and Glantz [34] found that the re-normalisation of smoking, mediated by the prevalence of e-cigarette use, can lead to a net negative impact on the health of young people. Our model sheds further light on the potential significance of the social influence of close peers and the broader community on individuals’ behavioural decisions concerning smoking.

The latest survey of tobacco use among students in middle school and high school in the United States found that, after a sudden drop in the prevalence of e-cigarette use in 2016, induced by the United States Food and Drug Administration’s new jurisdiction over the sale of e-cigarettes to people aged under 18 years, the prevalence of e-cigarette use among high school teenagers remained at around 12%. Furthermore, the decline in any kind of combustible smoking slowed down in 2017, compared with the drastic decrease in conventional tobacco smoking with the rapid expansion of e-cigarettes in 2014 [35]. Given the increasing prevalence of e-cigarettes in general, the social influence of an open attitude to e-cigarettes will become even stronger, and our simulation results imply that this enhanced influence may result in the stagnation of the declining trend in tobacco use among young people in the future [6].

The model used in the present study has several limitations. First, the social networks we constructed for young people in this study were conceptual and static throughout our simulations because of a lack of data on real-world youth network dynamics at the population level. For this reason, we focused more on influences at the level of the whole network and less on influences at the level of the local network. Future studies should examine these network effects in detail. Second, we developed our model based on the National Youth Tobacco Survey data in the United States, and, thus, any limitations in those data may also apply to our model. In particular, we used the Survey’s estimates of ‘current use’ (last 30 days) of conventional cigarettes or e-cigarettes, and we could not precisely discriminate ‘initiation’ from ‘regular use’, which may have resulted in biased estimation. In addition, our model cannot be applied beyond teenagers in high school in the United States; independent model development is required for different populations. Third, our simulation model does not incorporate social influence from sources other than the surrounding personal network. Recent studies have called attention to the increasing influence of commercial advertisements through the mass media and on the Internet, where e-cigarettes’ customisability and ability to accommodate personal modifications are emphasised to attract young people [35]. Fourth, although we relied on a utility-based model of smoking choice behaviour, recent neurological research on the reward reinforcement learning system provides an alternative basis for modelling the behavioural choice of nicotine substance use [36–38]. However, in our understanding, these previous studies explain how nicotine hinders smoking cessation by creating nicotine-induced rewards. Less research has mentioned how nicotine affects smoking initiation among adolescents. Some studies have indicated that social influence affects adolescents’ smoking behaviours via activating the reward system [39]. An experimental animal study with rats found that the interaction between social environment (peer existence) and nicotine uptake induces early gene expression in reward-related regions, suggesting that social influence enhances the neurobiological effect of nicotine by modifying the reward system to facilitate initiation and maintenance of nicotine preference [40]. Utility is an expression of reward valuation, and our model incorporates the utility function of smoking, which is affected by social influence and substance use experience. We believe that our model is not totally isolated from existing neurobiological findings on the mechanism linking nicotine to adolescents’ smoking behaviours. However, the integration of utility theory and the neurobiological basis of reward requires further study. Finally, a recent review of the application of agent-based modelling to public health issues argues that the model should incorporate a broader array of interrelated health conditions and behaviours, rather than narrowly focusing on one particular health behaviour [22]. Because of limited data availability, it was beyond our scope and capability to integrate behaviours other than smoking in the present study. With improved data availability, future studies could incorporate both smoking and other risky behaviours among adolescents to better capture the impact of social influence on adolescents’ quality of life as a whole.

To conclude, our simulation results using agent-based modelling indicate that an increase in the prevalence of e-cigarette use in the peer network and in the general adult population may exert social influence on teenagers in high school to initiate or continue conventional cigarette smoking. Assessing the impact of e-cigarettes in the general population as a ‘healthier’ alternative to conventional smoking may require careful monitoring of trends in young people’s smoking behaviours.

## Supporting information

S1 Appendix Rationale and technical details of the newly developed agent-based model. (DOCX)

S2 Appendix Simulation algorithm in NetLogo 5.3.1. (DOCX)

S1 Table Description of parameters in the simulation model. (XLSX)

S2 Table Initial statistics of the simulation parameters. (XLSX)
