# Supplementary material for: Social influence of e-cigarette smoking prevalence on smoking behaviours among high-school teenagers: Microsimulation experiments
Source: PLoS One. 2019 Aug 29;14(8):e0221557. doi: 10.1371/journal.pone.0221557 (PMC6715222; doi:10.1371/journal.pone.0221557)
Supplement: S1 Appendix — (DOCX) [file pone.0221557.s001.docx]

**Supporting Information File (S1)**

**Section 1. Agent-based modelling and rationale in this study**

Our study purpose in this manuscript, as is described in the text, is to quantitatively assess the impact of social influence from peers and from general population on adolescence behavioral decision to adopt smoking behaviors. Our specific focus is put on the social interactions between individuals (or agents) and their peers (or alters), and between agents and environment (e.g. smoking prevalence in general).

Our rationale to regard adolescence adoption of smoking behavior as a socially constructed phenomena was that previous surveys on adolescence smoking behavior depicted that their decision to initiate smoking is strongly affected by others’ behaviors and behavioral norms about smoking among their adolescence peers, parents, and more general behavioral trend in the society [refer to #4, 12, 14, 15, 17, and 20 in the maintext]. However, the magnitude of the social influence regarding e-cigarette smoking on adolescence conventional smoking behavior is not fully assessed quantitatively in existing literature despite of rapid prevalence and popularity of e-cigarette among the youth in the US. Our study intends to fill this knowledge gap.

By explicitly tracing complex processes of social interaction among adolescence, we ultimately aim at quantifying the impact of e-cigarette smoking prevalence onto smoking prevalence in adolescence population as a whole, through a bottom-up aggregation of individual smoking behaviors under social influences. Although the trend projection of smoking prevalence in the population level is also available in other modeling (e.g. regression with population level variables, or compartmental modeling such as system dynamics), they do not incorporate individual-individual and individual-environmental interactions that evolve over feedbacks and adaptations. Instead, we chose agent based modeling as more suitable among other available modeling methods because the model could explicitly incorporate a set of behavioral/interactional rules at individual level to causally explain the pattern of behaviors emerging in the population level over time [S1]

However, it is not our purpose to offer a universally applicable model of individual’s decisional mechanism for nicotine substance adoption. Instead, we used a simple plausible simulation model to conduct a counter-factual experiment to assess the impact of social influence on adolescence smoking behavior in individual level and subsequent prevalence trend in adolescence population level.

Agent based modeling is used to simulate individuals’ decision making according to a set of simple programmed rules of social interaction, which generates individual’s heterogeneity and also an organized behavior as a social group [S1][#22 in the main text]. By repeatedly running simulations with a range of parameters (in our case, “openness” and “cross-over” effect between e-cigarette smoking and combustible cigarette smoking), ABM would provide an insight for what would happen in adolescence smoking prevalence if their attitude toward smoking could be modified by vicarious observation of prevailing e-cigarette and cigarette smoking in the peer and the society in general, while keeping the other conditions the same.

Furthermore, we suspected if the prevalence of e-cigarette smoking, which is recommended as an effective tool for adult’s smoker to quit, could induce unintended consequences on youth smoking initiation. One advantage of the agent based modeling over traditional epidemiological analytic approaches is their ability to identify such unintended consequences emerging from complex social influence (ibid, pp81).

**Section 2. Technical details of developed agent-based model**

**2.1 Model overview**

Our model of smoking behavioral choice by individual adolescence is based on a utility function of choice, comprising of utility calculation to smoke or not conventional tobacco, e-cigarette, or to stay as non-smoker. We basically followed the utility function model under interactions between habits and social influences proposed in the socio-behavioral economics theory of rational addiction by Becker and Murphy (2000) [s2, s3].

The utility is assumed to be a function of habits depending on one’s past behavior and social influences from behaviors of others. Becker and Murphy argued that “a person is more likely to smoke if she has already engaged in these activities for a while.”(s3; pp17) More specifically, “habits” are expressed as a function of accumulated time duration of each smoking status (conventional tobacco smoker, e-cigarette smoker, and non-smoker statuses), with diminishing utility over time length [s2].

With this predetermined rules in individual levels, each adolescence (agent) is allowed to interact with other peer agents within her/his social network as is depicted in the next subsection 2.2. Each agent is assigned a baseline condition by random assignment to allow heterogeneity across agents. Through the interaction with other agents and vicarious observations of smoking popularity in the society as a whole, the agent’s utility function is revised, and the agent adopts a behavior based on the revised utility. These interaction processes are repeated to finally generate a group level trend of behaviors.

**2.2 Design of the agent network**

Our agent-based model contained a scale-free network of 3000 high-school teenager agents. We generated the network based on a preferential attachment model in which agents preferred to connect to other agents who already had more connections [s4]. More specifically, each of the agents had an average out-degree of five and was connected to and by an average of 10 other agents in total. We initially created a small basic random network with several agents. We then sequentially introduced additional agents to the network who were more likely to be connected to existing agents who already had more connections in the network. For example, when we assigned an agent to connect to 10 agents in the existing network, that agent was connected to a certain number (n) of “smoker” agents, according to their response to the question “How many of your four closest friends smoke cigarettes?” and (10 − n) “non-smoker” agents.

**2.3 Decision model development**

We determined the initial distribution of the smoking statuses of the agents by the percentage of current use (last 30 days) of conventional cigarettes or e-cigarettes among US high-school students based on the National Youth Tobacco Survey 2011 data [s5]. In each decision cycle, the agents evaluated their utility for the potential alternatives of smoking conventional cigarettes and/or e-cigarettes or remaining a non-smoker, chose the alternative with the highest utility, and updated their smoking status and perceived openness to smoking for the next cycle. To specify further, each agent had a utility function that took into account the benefits of conventional cigarette smoking, e-cigarette smoking, and remaining a non-smoker:

$U\left( {smokeCC}_{t} \right)=U\left( t_{cc},f\left( openessCC, n_{t},m_{t},crossover*{(k_{t,} + j}_{t,}) \right) \right)$ (1)

$U\left( {smokeEC}_{t} \right)=U\left( t_{ec},f\left( openessEC, k_{t,},j_{t,}, crossover*{{(n}_{t} + m}_{t}) \right) \right)$ (2)

$U\left( {smokeNone}_{t} \right)=U\left( t_{ns} \right)$ (3),

where $U\left( {smokeCC}_{t} \right)$ is the utility of smoking conventional cigarettes, $U\left( {smokeEC}_{t} \right)$is the utility of smoking e-cigarettes, and $U\left( {smokeNone}_{t} \right)$ is the utility of remaining a non-smoker.

The utility function for smoking included two large components: the agents’ own smoking experiences and the social influence of the vicarious experience of others. The agents’ own experiences were parameterized as:$t_{cc}$, related to the accumulated duration of smoking conventional cigarettes;$t_{ec}$ related to the accumulated duration of smoking e-cigarettes; and $t_{ns}$, related to the accumulated duration of being a non-smoker. We assumed that the marginal utility gain diminished as the time spent in a corresponding status increased.^3^

To conceptualize the social influence of others, we introduced agents’ “openness to smoking” or their tendency toward smoking as predisposing factors for the social influence of others. We quantified this tendency based on the responses to the following questions: “If one of your friends offered you a cigarette, would you smoke it?” and “Do you think you will smoke a cigarette anytime during the next year?” We then assumed that perceived social influence is shaped by agents’ perceptions of the social popularity of smoking gained by assessing smoking prevalence in their own direct network and in the United States in general.

Thus, $f\left( openessCC, n_{t},m_{t} \right)$ is the perceived social influence on conventional cigarette smoking derived from the conventional cigarette-smoking teenagers among the agents’ direct social contacts ($n_{t})$ and from conventional cigarette smokers in society as a whole $( m_{t}$), given the agents’ openness to conventional cigarette smoking at time *t*. Similarly, $f\left( openessEC, k_{t,},j_{t} \right)$ is the perceived social influence on e-cigarette smoking derived from e-cigarette-smoking teenagers among the agents’ direct social contacts $(k_{t})$and from e-cigarette smokers in society as a whole ($j_{t})$, given the agents’ openness to e-cigarette smoking at time *t*.

Furthermore, we incorporated $"crossover"$ to represent the possible influence of the prevalence of e-cigarette smoking on the social influence on conventional cigarette smoking, and vice versa. Thus, the prevalence of e-cigarette smoking in the local network ($k_{t})$and in society as a whole ($j_{t}$) affected the influence on conventional cigarette smoking, as shown in Formula (1). In the baseline scenario, we assumed that $"\mathrm{crossover}"=0$, indicating that the influences of e-cigarette and conventional cigarette smoking were independent.

Finally, the agents compared the obtained utilities and made decisions according to the following rules:

If$U\left( {smokeCC}_{t} \right)>U\left( {smokeNone}_{t} \right) \mathrm{and} U\left( {smokeEC}_{t} \right)>U\left( {smokeNone}_{t} \right) \mathrm{and} \mid U\left( {smokeCC}_{t} \right)- U\left( {smokeEC}_{t} \right)\mid<C,$ then *dual smoke.*

*(C is a small constant.)*

If$U\left( {smokeEC}_{t} \right)$ $> U\left( {smokeNone}_{t} \right) \mathrm{and} U\left( {smokeEC}_{t} \right) -U\left( {smokeCC}_{t} \right) \geq C,$then *smoke e-cigarettes.*

If$U\left( {smokeCC}_{t} \right)$ $>U\left( {smokeNone}_{t} \right) \mathrm{and} U\left( {smokeCC}_{t} \right) -U\left( {smokeEC}_{t} \right)\geq C,$then *smoke conventional cigarettes.*

Otherwise, *stay non-smoking.*

Then, $t_{cc} t_{ec} t_{ns}$ would be updated according to the above decision.

**2.3 Validation of the simulation model**

A model of agent based modeling is often viewed in isolation, and model validation is often conducted through examining the extent to which the model output approximates real-world data drawn from existing empirical research [s6]. Often used is the “backward validation” where a model simulates the past trend of a focused phenomenon, and compared the actual trend observed in the past real-world. In our case, we calibrated our model to fit to actual trend of adolescence smoking of combustible cigarette and e-cigarette derived from the National Youth Survey of Tobacco in the U.S. As we showed in the text, the calibrated model projection matched 95% confidence interval of actual trend of smoking prevalence (see Fig 1).

**References**

s1. Badham J, Chattoe-Brown E. Gilbert N, Chalabi Z, Kee F, Hunter RF. Developing agent-based models of complex health behaviour. Health Place 2018; 54:170-177. doi: 10.1016/j.healthplace.2018.08.022

s2. Becker GG, Murphy KM. A Theory of Rational Addiction. *J Polit Econ*. 1988;96(4):675-700. doi:10.1086/261558.

s3 Becker GG, Murphy KM. Social economics Harvard University Press, 1997

s4 Barabasi AL, Albert R. Emergence of scaling in random networks. Science 1999;286(October):509-12. doi:10.1126/science.286.5439.509.

s5 CDC’s Office on Smoking and Health. National Youth Tobacco Survey (NYTS). https://www.cdc.gov/tobacco/data_statistics/surveys/nyts/index.htm. Accessed May 2, 2018.

s6 Windrum P, Fagiolog G, Moneta A. Empirical Validation of Agent-Based Models; Alternatives and Prospects. J Artf Soc Social Sim. 2007; (2):8. http://jasss.soc.surrey.ac.uk/10/2/8.html
