# Supplementary material for: Social influence of e-cigarette smoking prevalence on smoking behaviours among high-school teenagers: Microsimulation experiments
Source: PLoS One. 2019 Aug 29;14(8):e0221557. doi: 10.1371/journal.pone.0221557 (PMC6715222; doi:10.1371/journal.pone.0221557)
Supplement: S2 Appendix — (DOCX) [file pone.0221557.s002.docx]

**Supporting Information File (S2)**

**Model algorithm with NetLogo 5.3.1**

extensions [csv matrix]

breed [human person]

globals [time tfses tmses gdppc smses sfses dses gdppcnow ecost ccost datao pp

minrts

maxrts

minerts

maxerts

minsts

maxsts

minests

maxests

minets

maxets

mincts

maxcts

minects

maxects

minhts

maxhts

lstcc

lstec

lstds

p1

p2

c1

c2

c3

c4

c5

c6

]

human-own [mark2 ua ue uh pt nf nfm opens opensm chara ps ties ses ses-s inf smoke dmses dfses addiction addict totaladdict erts statee etotaladdict hcts einf ects ests eaddict eaddiction health states stateh mark my-partners1 my-partners2 esmoke utility rts sts ets cts]

;;The preferential attachment algorithm,layout algorithm are adapted from the “preferential attachment” model in netlogo library

;;Wilensky, U. (2005). NetLogo Preferential Attachment model. http://ccl.northwestern.edu/netlogo/models/PreferentialAttachment. Center for Connected Learning and Computer-Based Modeling, Northwestern University, Evanston, IL.

;;Wilensky, U. (1999). NetLogo. http://ccl.northwestern.edu/netlogo/. Center for Connected Learning and Computer-Based Modeling, Northwestern University, Evanston, IL.

;;;;;;;;;;;;;;;;;;;;;;;;

;;; Setup Procedures ;;;

;;;;;;;;;;;;;;;;;;;;;;;;

to setup

clear-all

set lstcc matrix:make-constant 100 13 999

set lstec matrix:make-constant 100 13 999

set lstds matrix:make-constant 100 13 999

random-seed seed

set seed2 0

create-agents ;;setup agents and networks

setup-parameters ;;setup ses, addction

random-seed seed2

reset-ticks

end

to create-agents

set datao []

set datao but-first (csv:from-file "xx2011.csv" ";")

let tt 0

while [tt < 4] [

create-human ( 1 + round (item 4 item tt datao / 3 / item 2 item tt datao )) [

set chara item tt datao

set opens round (random-normal item 2 chara item 3 chara)

if opens < 1 [set opens 1]

if opens > 4 [set opens 4]

set nf round (random-normal item 5 chara item 6 chara)

if nf < 0 [set nf 0]

if nf > 4 [set nf 4]

set ses 5

set smoke item 0 chara

set esmoke item 1 chara

set opensm 1

if opens > 3 [set opensm 0]

set opens opens * (( 1 + random 25 ) / 100 )

]

set tt tt + 1 ]

ask human [if smoke = 2 [set smoke 0]

if esmoke = 2 [set esmoke 0]

rt random 360

fd random 50

set size 1

]

layout

ask human

[ let tties 0

let t random 100

if t <= 10 [ set tties 1]

if t > 10 and t <= 25 [set tties 2]

if t > 25 and t <= 41 [set tties 3]

if t > 41 and t <= 61 [set tties 4]

if t > 61 and t <= 76 [set tties 5]

if t > 76 and t <= 94 [set tties 6]

if t > 94 [set tties one-of [7 8 9 10 11] ]

set mark 0

set ties 5

]

ask n-of ( floor (0.05 * count human) ) human [

set mark 1]

ask human with [mark = 1] [

create-links-to min-n-of nf human with [who != [who] of myself and mark = 1 and smoke = 1][abs (opens - [opens] of myself)]

if ties > nf [create-links-to n-of (ties - nf ) human with [who != [who] of myself and mark = 1 and smoke = 0]]

]

let r 0

while [r < count human - count human with [ties = 0]- floor (0.05 * count human)] [ask max-one-of human with [mark = 0 and ties > 0][ties][

create-links-to min-n-of nf human with [who != [who] of myself and mark = 1 and smoke = 1][abs (opens - [opens] of myself)]

if ties > nf [ create-links-to min-n-of (ties - nf ) human with [who != [who] of myself and mark = 1 and smoke = 0][abs (opens - [opens] of myself)] ]

set mark 1

]

set r r + 1

]

;;clear-links

;;ask human [ create-links-to n-of ties other human ]

ask human with [count in-link-neighbors + count out-link-neighbors = 0] [create-links-to n-of ties human with [who != [who] of myself]]

ask links [hide-link]

ask human [if count out-link-neighbors with [smoke = 1] > 0 [set nfm 1]]

end

to setup-parameters

ask human [ set ses-s (ties ) / 10 ]

;;set up addiction, health

ask human with [smoke = 1][set addiction (1 + random 9 ) ]

ask human with [esmoke = 1][set eaddiction (1 + random 9 ) ]

ask n-of round (0.005 * count human ) human with [smoke = 0][set smoke 1 set addiction (1 + random 9 ) ]

ask human [set addiction addiction / 10

set eaddiction eaddiction / 10

if addiction + eaddiction > 1 [set eaddiction 1 - addiction]

set states addiction / 0.0001

set statee eaddiction / 0.0001

]

ask human [set health 1 - addiction - eaddiction

if health < 0

[ set health 0]

set stateh health / 0.0001

]

ask human [set my-partners1 out-link-neighbors

set my-partners2 in-link-neighbors

set ps count my-partners1 with [smoke = 1] + count my-partners2 with [smoke = 1]

]

set pp count human

end

;;;;;;;;;;;;;;;;;;;;;;;

;;; Main Procedures ;;;

;;;;;;;;;;;;;;;;;;;;;;;

to go ;;repeat

if (ticks mod 12 = 0)[

matrix:set lstcc seed2 0 (count human with [smoke = 1] / count human * 100)

matrix:set lstec seed2 0 (count human with [esmoke = 1] / count human * 100)

matrix:set lstds seed2 0 (count human with [smoke = 1 and esmoke = 1] / count human * 100)]

decide-smoke

tick

if ticks > 0 and (ticks mod 12 = 0) [

set seed2 seed2 + 1

random-seed seed

clear-turtles

create-agents ;;setup agents and networks

setup-parameters ;;setup ses, addction

random-seed seed2

reset-ticks

]

end

to decide-smoke

let skp count human with [smoke = 0 and esmoke = 0] / count human * 100

let eskp count human with [esmoke = 1 and smoke = 0] / count human * 100

ask human [

set addict addiction * ses-s

set eaddict eaddiction * ses-s

set inf sum [ses-s ] of my-partners1 + sum [ses-s] of my-partners2

set totaladdict sum [addict] of my-partners1 + sum [addict] of my-partners2

set einf inf

set etotaladdict sum [eaddict] of my-partners1 + sum [eaddict] of my-partners2

set einf inf

set rts totaladdict / inf

set erts etotaladdict / einf

set sts ( 1 - e ^ ( - 0.01 * states))

set ests ( 1 - e ^ ( - 0.01 * statee))

set ets ( 1 - e ^ ( - 0.01 * stateh))

set hcts health

set cts (p1 - p2 * opens) * addiction

set ects (p1 - p2 * opens) * eaddiction

]

if ticks = 0[

set minrts [rts] of min-one-of human [rts]

set maxrts [rts] of max-one-of human [rts]

set minerts [erts] of min-one-of human [erts]

set maxerts [erts] of max-one-of human [erts]

set minsts [sts] of min-one-of human [sts]

set maxsts [sts] of max-one-of human [sts]

set minests [ests] of min-one-of human [ests]

set maxests [ests] of max-one-of human [ests]

set minets [ets] of min-one-of human [ets]

set maxets [ets] of max-one-of human [ets]

set mincts [cts] of min-one-of human [cts]

set maxcts [cts] of max-one-of human [cts]

set minects [ects] of min-one-of human [ects]

set maxects [ects] of max-one-of human [ects]

set minhts [hcts] of min-one-of human [hcts]

set maxhts [hcts] of max-one-of human [hcts]

]

ask human [

if ticks = 0 [

set rts ( rts - minrts ) / ( maxrts - minrts)

set erts ( erts - minerts ) / ( maxerts - minerts)

set sts ( sts - minsts ) / ( maxsts - minsts)

set ests ( ests - minests ) / ( maxests - minests)

set ets ( ets - minets ) / ( maxets - minets)

set cts ( cts - mincts ) / ( maxcts - mincts)

set ects ( ects - minects ) / ( maxects - minects)

set hcts ( ects - minects ) / ( maxects - minects)

]

set ua ( c1 - opens) * ( rts + cts + sts ) - c2 * ticks ^ c3

set uh ( ets + hcts )

set ue ( c1 - opens) * ( erts + hcts + ects + ests ) + c4 * eskp ^ c3 + (c5 - ticks ) * c6

if ua > uh and ue + 0.15 > uh [set states states + 1

set statee statee + 1

set addiction addiction + 1.5

set eaddiction eaddiction + 0.5

set smoke 1

set esmoke 1

set mark2 1

]

if uh >= ue + 0.15 and uh >= ua [

set mark2 1

set stateh stateh + 1

set health health + 1

set smoke 0

set esmoke 0

]

if mark2 = 0

[ if max (list ua uh ue ) = ua [

set states states + 1

set addiction addiction + 1.5

set smoke 1

set esmoke 0

]

if max (list ua uh ue ) = ue

[ set statee statee + 1

set stateh stateh + 1

set opens opens - 0.01

set eaddiction eaddiction + 0.5

set health health + 1

set smoke 0

set esmoke 1

]

]

]

ask human [set mark2 0]

matrix:set lstcc seed2 (ticks + 1) (count human with [smoke = 1] / count human * 100)

matrix:set lstec seed2 (ticks + 1) (count human with [esmoke = 1] / count human * 100)

matrix:set lstds seed2 (ticks + 1) (count human with [smoke = 1 and esmoke = 1] / count human * 100)

end

;;;;;;;;;;;;;;

;;; Layout ;;;

;;;;;;;;;;;;;;

to layout

;; the number 3 here is arbitrary; more repetitions slows down the

;; model, but too few gives poor layouts

repeat 3 [

;; the more human we have to fit into the same amount of space,

;; the smaller the inputs to layout-spring we'll need to use

let factor sqrt count human

;; numbers here are arbitrarily chosen for pleasing appearance

layout-spring human links (1 / factor) (7 / factor) (10 / factor)

display ;; for smooth animation

]

;; don't bump the edges of the world

let x-offset max [xcor] of human + min [xcor] of human

let y-offset max [ycor] of human + min [ycor] of human

;; big jumps look funny, so only adjust a little each time

set x-offset limit-magnitude x-offset 0.1

set y-offset limit-magnitude y-offset 0.1

ask human [ setxy (xcor - x-offset / 2) (ycor - y-offset / 2) ]

end

to-report limit-magnitude [number limit]

if number > limit [ report limit ]

if number < (- limit) [ report (- limit) ]

report number

end
